# Attitudes, current behaviours and barriers to public health measures that reduce COVID-19 transmission: A qualitative study to inform public health messaging

**DOI:** 10.1371/journal.pone.0246941

**Published:** 2021-02-19

**Authors:** Jamie L. Benham, Raynell Lang, Katharina Kovacs Burns, Gail MacKean, Tova Léveillé, Brandi McCormack, Hasan Sheikh, Madison M. Fullerton, Theresa Tang, Jean-Christophe Boucher, Cora Constantinescu, Mehdi Mourali, Robert J. Oxoby, Braden J. Manns, Jia Hu, Deborah A. Marshall

**Affiliations:** 1 Department of Medicine, Cumming School of Medicine, University of Calgary, Calgary, AB, Canada; 2 Department of Community Health Sciences, Cumming School of Medicine, University of Calgary, Calgary, AB, Canada; 3 Data & Analytics, Alberta Health Services, Calgary, AB, Canada; 4 School of Public Health, University of Alberta, Edmonton, AB, Canada; 5 Department of Family and Community Medicine, University of Toronto, Toronto, ON, Canada; 6 School of Public Policy and Department of Political Science, University of Calgary, Calgary, AB, Canada; 7 Haskayne School of Business, University of Calgary, Calgary, AB, Canada; 8 Department of Economics, Faculty of Arts, University of Calgary, Calgary, AB, Canada; Middlesex University, UNITED KINGDOM

## Abstract

Public health measures to reduce COVID-19 transmission include masking in public places, physical distancing, staying home when ill, avoiding high-risk locations, using a contact tracing app, and being willing to take a COVID-19 vaccine. However, adoption of these measures varies greatly. We aimed to improve health messaging to increase adherence to public health behaviours to reduce COVID-19 transmission by: 1) determining attitudes towards public health measures and current behaviours; 2) identifying barriers to following public health measures; and, 3) identifying public health communication strategies. We recruited participants from a random panel of 3000 phone numbers across Alberta to fill a predetermined quota: age (18–29; 30–59; 60+ years), geographic location (urban; rural), and whether they had school-age children. Two researchers coded and themed all transcripts. We performed content analysis and in-depth thematic analysis. Nine focus groups were conducted with 2–8 participants/group in August-September, 2020. Several themes were identified: 1) importance of public health measures; 2) compliance with public health measures; 3) critiques of public health messaging; and 4) suggestions for improving public health messaging. Physical distancing and masking were seen as more important than using a contact tracing app. There were mixed views around willingness to take COVID-19 vaccine. Current public health messaging was perceived as conflicting. Participants felt that consistent messaging and using social media to reach younger people would be helpful. In conclusion, these findings provide insights that can be used to inform targeted (e.g., by age, current behaviour) public health communications to encourage behaviors that reduce COVID-19 transmission.

## Introduction

As of December 2020, there have been more than 442,000 confirmed cases of COVID-19 in Canada, with over 75,000 in Alberta [1]. Public health measures are critical to reducing transmission [2–8]. These public health measures include wearing face masks, physical distancing, staying home when ill, avoiding high-risk spaces like crowded indoor gatherings, using contact tracing apps and being willing to take a vaccine when available [9].

A national poll of Canadians conducted by the Angus Reid Institute in August 2020, six months into the global COVID-19 pandemic, identified that Canadians had three general mindsets related to COVID-19 prevention [10]. They found that approximately half of Canadians were “infection fighters” who consistently follow public health measures, just over one-third were “inconsistent”–following some public health measures but not others, and one-fifth were “cynical spreaders” who disregard most public health measures. A survey in September 2020, found that 60% of Canadians had relaxed one or more public health measures in the previous month [11]. There also appears to be declining willingness to take a vaccine–in July, 46% of Canadians would take a vaccine as soon it was available, a figure which declined to 39% in September [12].

As Canada experiences its “second wave” combined with growing reluctance by governments to impose mandatory measures like lockdowns, voluntary behaviour change is critical to reducing transmission. The Theoretical Domains Framework [13] tells us that in order to facilitate behaviour change, we need to understand the characteristics of the people in whom we are trying to effect change, their behavioural context, and the components driving change. Therefore, understanding current attitudes and behaviours as well as barriers to adopting public health measures is critical in developing effective public health communication designed to change behaviour and reduce transmission of COVID-19. Strategies from social and behavioural science can then be used to encourage behaviour change [14].

The overall objective of our study was to identify ways in which public health messaging around COVID-19 mitigation could be improved to facilitate adherence to public health behaviours. To accomplish this, this study seeks: 1) to determine Albertans’ attitudes towards public health measures and current behaviours including wearing face masks in public spaces, physical distancing, staying home when ill, avoiding high-risk spaces like crowded indoor gatherings, using contact tracing apps and being willing to take a vaccine when available; 2) to elucidate barriers to following public health behaviours; and 3) to identify public health communication strategies to facilitate behaviour change.

## Materials and methods

### Study design and population

We conducted focus groups with targeted populations in Alberta, Canada between August 27 and September 10, 2020. The study was approved by the University of Calgary Conjoint Health Research Ethics Board (REB20-1228). Participation was voluntary and written informed consent was obtained.

### Participant recruitment

Nine focus groups were planned (Table 1), including seven from the areas in Alberta with the highest COVID-19 case counts, Calgary, Edmonton, and other urban areas. Within the four Calgary focus groups, two were for individuals aged 18–29 years, and one each for ages 30–59 years and 60+ years. Three focus groups were planned for residents from Edmonton and other urban areas (one for each age group: 18–29 years; 30–59 years; and 60+ years). One focus group included only rural Alberta residents of any age. As the focus groups occurred when schools were reopening, one focus group was planned for parents with school-age children.

**Table 1:** Focus group characteristics for Alberta, Canada.

Focus Group ID	Demographic	Age category (years)
1	Calgary	18–29
2	Calgary	18–29
3	Calgary	30–59
4	Calgary	60+
5	Edmonton and Other Urban Areas*	18–29
6	Edmonton and Other Urban Areas*	30–59
7	Edmonton and Other Urban Areas*	60+
8	Rural	18+
9	Parents with school age children	18+

*Other urban areas included Fort McMurray, Red Deer, Medicine Hat, Lethbridge and Grand Prairie.

A random sample of potential participants was contacted by phone using a purchased telephone sample of 3000 residential geocoded landline and cellphone numbers of Alberta residents. The telephone population sampling frame was built using random digit dialing methodology and Interactive Voice Response was used to confirm the numbers were active. Participants were considered for inclusion if they were Alberta residents aged 18 years or older, spoke English and had internet access. Focus group participants were selected using a quota system according to age, sex, geographic location, whether they had school-age children, and adherence to public health measures (wearing face masks, physical distancing, staying home when ill, avoiding high-risk spaces, and use of contact tracing apps). We aimed to make the groups heterogeneous with respect to adherence to public health measures. Individuals who completed the screening survey were invited to complete an online consent form and respond to questions that would align with one of the specific targeted focus groups. A small incentive was offered for participation.

### Focus group guide development

A literature review [15] was conducted to determine known attitudes and current health behaviours to public health measures to direct the content of the focus groups. In addition, expert and clinical stakeholder input was sought from the Chief Medical Officer of Health of Alberta, Alberta Health (the Ministry of Health), and Alberta Health Services.

The focus group content centered around attitudinal and behavioural measures (e.g., risk preferences, social attitudes), knowledge of COVID-19, and knowledge and attitudes towards public health strategies. This content was collated in the form of a semi-structured interview guide with six key questions targeting the public health measures for use in the focus groups (S1 File).

### Focus group moderation

Alberta Health Services Analytics conducted focus groups to data saturation (i.e., the addition of participants did not reveal new emergent themes) [16]. Focus groups were 1.5 hours long and were done online with Zoom (Zoom Video Communications, Inc., San Jose, CA) due to COVID-19 restrictions. The aims of the study were reviewed at the beginning of each focus group. Each focus group session was led by one of three skilled female moderators while assistant moderators observed and took notes. The facilitators did not share their personal attitudes and behaviours. A team debrief (only those formally invited) was held after each focus group session, and field notes were documented. There were no repeat interviews.

### Analysis

The online focus group discussions were video recorded and transcribed verbatim. Content analysis was conducted to identify themes followed by in-depth thematic analysis to identify common perceptions and opinions. A qualitative data analysis software, NVivo Qualitative Data Analysis Software (QSR International, Version 12) was used to support data organization and analysis. A preliminary analytic template, aligned with the focus group guide, was developed as a starting point for analysis. Two experienced qualitative data analysts did the initial coding of the transcripts, with the analytic template continuing to evolve throughout the course of the data analysis. Regular communication between the two analysts ensured that ongoing changes to the template were discussed and agreed upon. Triangulation of themes and codes was also done by reviewing field notes recorded during each focus group, and checking the emergent findings with the facilitators of the focus groups to ensure no key themes were missed. Participants did not provide feedback nor review transcripts. The consolidated criteria for reporting qualitative research (COREQ) checklist [16] was used to report our findings.

## Results

Of 1,635 potential focus group participants contacted, 60 (3.7%) completed the online screening process and were invited to participate in a focus group. Of those 60 potential participants, 50 participants attended across nine focus groups, while 10 did not attend. Focus group size ranged from 2 to 8 participants. Overall, the groups included 20 (40%) males and 30 (60%) females. The ages of the participants were distributed as follows: 17 (34%) age 18–29 years, 6 (12%) age 30–39 years, 4 (8%) age 40–49 years, 10 (20%) age 50–59 years, and 13 (26%) age 60 years or older. Current behaviours with respect to each of the public health measures explored in this study are presented in Table 2.

**Table 2:** Current behaviours with respect to public health measures to reduce COVID-19 transmission.

PUBLIC HEALTH MEASURE	FINDINGS	QUOTES
Wearing Face Masks in Public Places	• Situations where mask wearing were more likely: ○ Any indoor public space ○ When physical distancing is not always possible, including outdoors ○ Where wearing a mask is mandatory	“We’re really getting mixed messages about masks, although for now, because it’s an easy thing for me to do, I do use them when I go to the store, or where I can’t social distance.”—Participant 41, Focus Group 7, 60+ year old female
	• Situations where mask wearing were less likely: ○ When eating or drinking ○ At larger family gatherings, where mask wearing may deemed to be culturally inappropriate ○ When it is not mandatory (e.g. a small town) ○ When unable to do so for medical or health reasons ○ Sometimes people forget to bring their masks with them, so will run a brief errand in an indoor public setting without a mask	
		“There are situations where 100 percent I don’t feel like wearing a mask, and I don’t do it. And maybe–and I know that’s not necessarily the best thing to do, but I know also when its 30 degrees out and I’m walking to a lineup, I don’t necessarily want to wear a mask for longer than I necessarily have to.”- Participant 26, Focus Group 2, 18–29 year old female
Physical Distancing	• Situations where physical distancing was more likely: ○ When interacting with individual outside of family or social bubbles ○ If members of their social bubble were more susceptible to COVID ○ When planning social gatherings with friends not in their bubble ○ When structural factors are in place to facilitate physical distancing (e.g., Plexiglass shields)	“My friends…we sort of pick our circle, but we keep it pretty limited, and there’s an understanding that each of us is going to be safe, we’re still going to see each other and we might, you know, exchange a high five or a hug, but we’re–we’re all doing the–the precautions that are mentioned: the masks, the sanitizer. I’m going plug that app so hard. Like just general hygiene.”–Participant 1, Focus Group 1, 18–29 year old female
	• Situations where physical distancing was less likely: ○ With members of family, friends or others in their social bubble (if they had one) ○ When outdoors ○ When using precautions (e.g., face mask, hand sanitizer)	
Staying Home When Ill	• Situations where people were more likely to stay home: ○ If experiencing minor symptoms (e.g., runny nose) ○ A few would stay home if really ill	“So come winter if I actually get a real flu, am I going to still be able to do my job? Yes, am I going to suffer while doing it?…If I don’t get paid, I don’t have an option…so yes I will go to work if I have to. With a mask, using as many precautions as I can, but unfortunately that’s the way my life is right now…I don’t think I’m alone in that.”–Participant 50, Focus Group 6, 30–59 year old female
	• Situations where people were less likely to stay home: ○ Working in a job that could not be done from home with little or no sick time ○ When something needed to be done that would prevent them from staying home (e.g., having an appointment, getting groceries, caring for a family member)	
Avoiding High-Risk Spaces	• Situations where people were more likely to go out: ○ With their social bubbles at socially distanced tables ○ At times, or to places, that are less busy ○ If establishments were following public health guidelines	“The regulations at restaurants and bars are so restricted already, that I–and they’re also following every single one of the guidelines. . . So I feel safe-ish, especially on a patio or something.”- Participant 4, Focus Group 1, 18–29 year old female
	• Situations where people were less likely to go out: ○ When the risk of contracting COVID is perceived to be too high (e.g., high case counts)	
Contact Tracing Apps	• A number of people had downloaded a contact tracing app • Some were planning to download the national tracing app, COVID Alert, when active in Alberta • Some had difficulties with downloading the app, or were not “tech savvy”	“Honestly…I like the sort of level of normalcy we have, so I’m pretty much ready to jump on board with any sort of measure [alluding to the app] that is easier than like social distancing.”–Participant 11, Focus Group 5, 18–29 year old male

### Wearing face masks in public places

Many participants felt that wearing face masks in public places where physical distancing was not possible was a way to protect others, was easy for most people to do and was an inexpensive way to reduce the spread of COVID-19. There were mixed views on whether face masks should be mandatory in indoor public spaces. Some participants noted that authorities have demonstrated poor role-modeling for face mask use in some settings.

“It drives me absolutely up the wall when I see people not wearing a mask, because it just—to me it just is the most selfish thing you can do …I don’t really wear the mask for me, I’m young I don’t have any underlying health problems, but I wear it for all of the people around me that might…get really sick from me, if I’m carrying it.”- Participant 3, Focus Group 1, 18–29 year old female

A number of barriers to wearing face masks were identified by participants including that masks may be difficult for some individuals to wear due to medical reasons, and that they might make communication difficult. Some participants noted that it can be difficult to convince others that they should wear a mask. Other identified barriers included cost, difficulty with enforcement, and lack of education around how to wear a mask properly (e.g., nose and mouth should be covered).

“They could have given us more information with regard to how to wear a mask… It seems like the general public doesn’t know all of these issues.”–Participant 36, Focus Group 7, 60+ year old female

### Physical distancing

Participants felt that concept of physical distancing was clear and understood how it prevents spread of COVID-19. They felt that most people practiced physical distancing when they could because it makes sense and is easy to do. Some specified that it will only work if those around you are also physical distancing, and a few participants said that they try to avoid social situations where physical distancing is not possible.

“It takes two people to follow the actual physical distancing rules, right. As much as you want to follow them and as much as…you want to stay away from people and keep that two meters between … you and another person, it’s all depends on the other person as well, right.”–Participant 6, Focus Group 1, 18–29 year old male

Participants reported experiencing difficulty performing physical distancing in busy, public, indoor settings such as grocery stores, shopping malls, and elevators. Workplaces were also identified as areas where physical distancing may be challenging. Examples of difficulties distancing within the workplace cited were carpooling in work vehicles to different work sites, and working in daycare or school settings. Some participants expressed concern that some people do not seem to either care or be aware of physical distancing.

“…our world hasn’t been designed really to keep everybody…two meters apart from each other, which I mean it sucks in hindsight…that wasn’t a thing that we had been thinking about beforehand. So that’s where a lot of the challenge comes in.”–Participant 12, Focus Group 5, 18–29 year old male

### Staying home when ill

“…now I’m a little bit more cautious if I was really sick, I would probably stay home just for the wellbeing of others. But normally I have to not be able to stand to not leave the house.”–Participant 10, Focus Group 5, 18–29 year old male

Many realized the importance of staying home when ill to protect others, although reported that communication around staying home when ill from authorities was unclear. Specifically, there was confusion about when people could return to work after being ill. Many participants felt that one has to trust people to do the right thing.

A common barrier discussed was that it was difficult to distinguish what “sick” really means in the context of COVID-19. Participants felt that the signs and symptoms of COVID-19 communicated by authorities were unclear, and that it was particularly difficult to identify symptoms of COVID-19 for those living with chronic conditions who regularly experience symptoms consistent with COVID-19 like a runny nose or cough. An unintended consequence of public health messaging discussed in the focus groups is that persons living with chronic conditions (e.g., allergies) can experience stigma when out in public.

“…it’s actually, you know difficult to distinguish, I think from personal experience, you know whether you have COVID, or whether you just have a cold, or some other kind of flu, right.”–Participant 58, Focus Group 9, 50–59 year old male

Staying home when ill was felt to be difficult for persons who have to go to a workplace compared with those who can work from home. In addition, there were mixed perspectives on whether people had enough sick time to stay home from work whenever they had a symptom consistent with COVID-19. Some felt that there is always enough sick time, while others reported not having any or enough sick time to follow public health measures.

### Avoiding high-risk spaces

Participants were asked about attending spaces such as bars, pubs, night clubs and lounges. The perception of the risk and risk tolerance varied considerably, although many recognized that there was some risk in going out to these places.

“…every individual has to manage their own risk and what they’re comfortable with…it’s a matter of managing yourself and what you’re comfortable with; and your own risk.”–Participant 62, Focus Group 9, 30–59 year old female

Some participants reported feeling safer in restaurants and pubs where good public health measures were in place compared with grocery stores and schools. Many participants, particularly those aged 18–29 years, felt that private parties were the riskiest type of gathering place, as there were often no public health measures in place.

“…they reopened the city back up… ourselves and some friends would go out, and again, weeks would go by no ill effects that we were aware of.”–Participant 21, Focus Group 3, 30–59 year old male

Despite the inherent risk, many participants expressed a desire to support businesses so that they would survive the pandemic.

### Using contact tracing apps

Participants were asked about the Alberta app, ABTraceTogether [17], and the Canada app, COVID Alert [18]. More participants were aware of the Canada-wide app than were aware of the Alberta app, and many felt that a Canada-wide app would be better than a province-specific one. Some saw contact tracing apps as a good tool for preventing spread, and felt it would be helpful to know if one had been exposed, both for oneself and for others. Some felt that younger people would benefit most, as they tend to go out more.

“I haven’t seen as much–I call it like advertising, but not really advertising, just I guess advocacy for the app. I haven’t seen nearly as much about that as I feel that we should, so I think there’s actually a lot of people that aren’t really aware of it to begin with.”–Participant 3, Focus Group 1, 18–29 year old female

The main concerns around using a contact tracing app were with respect to privacy and security in the older age groups. This was less of a concern for individuals aged 18–29 years. Some participants also were concerned about government tracking and surveillance, which was due in part to misconceptions about how the apps work.

“I have concerns about how much of my information is out there. And I think I’m not alone in that respect…So I, I would not be downloading the app.”–Participant 39, Focus Group 7, 60+ year old male

### Taking a COVID-19 vaccine

Participant responses to whether they would take a COVID-19 vaccine were mixed. Some participants across all age groups felt they would get the vaccine right away, while other participants said they would not take a COVID-19 vaccine feeling that COVID-19 would not affect their health or the health of their family members. Some participants, particularly in the older age groups, reported that they would be willing to take a vaccine but not right away, instead they would wait for further scientific evidence on vaccine safety and efficacy.

“I think we need a vaccine because like [name] was saying, you need to restart to live like normal.” Participant 27, Focus Group 2, 18–29 year old male

Participants felt that a vaccine could enable people to get back to their normal lives, and that it could be used to protect oneself and others. Many participants who reported that they regularly take the annual flu vaccine felt they would take a COVID-19 vaccine when available. A few felt that when a safe and effective vaccine is available, it should be mandatory for everyone to take it.

“…it’s kind of about protecting yourself and everybody around you.”–Participant 13, Focus Group 5, 18–29 year old female

Several barriers to vaccine uptake were discussed including a lack of confidence that a vaccine will work, and that it would not do harm. A few participants mentioned that they had experienced side effects with the annual flu shot (e.g., getting sick) and this would make them less likely to get a COVID-19 vaccine.

“I think that one thing that’s going to impact that uptake of vaccination here, is us hearing all about how the US is sidestepping their normal routines and their normal safety reviews, to push through a new vaccine… Even though Health Canada is much more cautious in their approval, I think that people will be scared off…of the safety aspects of this new vaccine.”–Participant 51, Focus Group 6, 30–59 year old female

### Additional themes

In addition to the themes emerging related to attitudes, behaviours and barriers to each public health measure, other themes were identified: 1) importance of public health measures; 2) compliance with public health measures; 3) critiques of public health messaging; and 4) suggestions for improving public health messaging.

### Importance of public health measures

Participants felt the most important measures for reducing transmission were physical distancing and masking. Participants preferred physical distancing over other public health behaviours because they felt distancing had the strongest scientific backing and was less onerous than other behaviours. Participants felt the contact tracing app was less important due to adoption being too low (too few downloads) and considering contact tracing a secondary measure (i.e., if everyone followed public health measures—distancing, masking—low community spread would not require contact tracing). Some participants felt a COVID-19 vaccine, if safe and effective, would be a good tool, in part because it would be covered under Alberta Health.

“…the ones that were deemed more important were, were things that were like backed by science, and I’m a huge advocate for science.”- Participant 4, Focus Group 1, 18–29 year old female

### Compliance with public health measures

Wearing face masks in public places was generally felt to be the easiest public health measure to adopt. It was noted that there may be challenges for school-age children to ensure that masks are available daily due to potential costs from lost masks and cleaning reusable masks. Physical distancing was felt to be easy to comply with for many with the exception of a few settings (e.g., school, private parties). Compliance with public health measures was felt to differ across age groups with participants from all age groups expressing that younger people are more likely to congregate in larger groups of friends. Interestingly, younger people indicated that older people were less likely to wear masks properly or physically distance.

“The hardest thing is just to say distance with friends, like people just like to hang out with people; that’s just all it is. Realistically at the end of the day, they go out with friends, whether it be big groups or smaller groups, people are hanging out with other people. That’s all it is.”—Participant 2, Focus Group 1, 18–29 year old male

### Critiques of public health messaging

The most common critique of the public health measures was the conflicting nature of the measures, with some saying it “does not make sense”. For example, some participants mentioned that public health guidelines were sending inconsistent messages by suggesting that physical distancing of 2 metres was required in some settings, but not in others such as in schools or on public transit. Some felt more resources were necessary to effectively implement public health measures, such as providing free or inexpensive masks or more funding to support distancing in schools. There were also criticisms about the politicization of some of the public health measures with many expressing that the measures should come from the Chief Medical Officer of Health rather than elected officials.

“I didn’t trust, the government’s position in terms of, of the mixed messaging that we receive.”- Participant 57, Focus Group 9, 30–39 year old female

### Suggestions for improving public health messaging

The participants provided several suggestions for how to change behaviours to mitigate COVID-19 and how to improve public health messaging in general and to each of the public health measures explored in the focus groups (Table 3). Overall, participants described COVID-19 public health communication to date as inconsistent, and suggested that messaging could be improved by framing it in three ways: 1) framing around protecting others rather than yourself; 2) providing more scientific or public health rationale for the importance of these behaviours; and 3) highlighting the importance of these behaviours for society to return to normal. Participants also suggested that tailored messaging strategies targeted at specific population segments like younger people should be explored.

**Table 3:** Focus group participant suggestions for how to change behaviour with respect to public health measures.

PUBLIC HEALTH MEASURE	FOCUS GROUP PARTCIPANT SUGGESTIONS FOR BEHAVIOUR CHANGE	QUOTES
All Measures	• Provide clear, consistent messaging • Increase awareness through advertising, advocacy, education, and information provided • Articulate that behaviours could lead to a return of normalcy • Provide better rationale/evidence for ‘why’ public health measures are important • Frame public health measures around protecting others and not only yourself • Make information easily accessible • By-laws may be necessary for some measures • Use social media and gamification, particularly if there is a desire to improve communication to young people	“So I think if we just advocated for it more, it would at least get people–more people aware of it [the contract tracing app], and then as a result increase the amount of people that subsequently download it.”- Participant 3, Focus Group 1, 18–29 year old female
		“We don’t have that kind of time, so the–the bylaws will be necessary because at this point its uh, well you’re not wearing a mask, we won’t serve you and you have to leave. And you, you can’t have any fun.”- Participant 1, Focus Group 1, 18–29 year old female
Wearing Face Masks in Public Places	• Make mask wearing mandatory through a by-law or business-led initiative and enforce it • More people wearing face masks would influence others to do the same • Provide more information about how to wear a face mask and differences in quality of masks	“I wear the mask—I’m in Edmonton so it is mandated here…I mean if you’re going to get people to wear it, probably it does have to be a bylaw…I wore it a little bit before the bylaw, mostly just because I was already doing it at work, so you know at that point I was used to it, and it wasn’t such an um, imposition.”- Participant 11, Focus Group 5, 18 = 29 year old male
Physical Distancing	• Public health officials need to provide clear, consistent information about the limitations of masks, and that physical distancing should still be practiced while wearing a mask • If there were more cases, this might influence behaviour	“I think it would be more instances of COVID…there’s not a huge fear factor with people until more cases are coming up.”–Participant 33, Focus Group 8, 30–59 year old female
Staying Home When Ill	• A culture shift is needed around going to work when ill, and while it has started, it is still going to be hard to convince some people to stay home • Ensure people have enough sick time, if they are unable to work from home • Ensure people have the proper supports, like running urgent errands or getting groceries	“So to me, I understand the practice, but I think a lot of clarification around the practice needs to be cleared up, particularly with employers, so that they understand what they need to do in order to be safe for their company, but at the same time protect their employees so that they can basically survive.”–Participant 52, Focus Group 6, 30–59 year old female
Avoiding High-Risk Spaces	• Most felt it would be hard to convince people to visit bars and pubs because social contact is important • Some noted that people might be influenced by peer pressure, outbreaks, or increased cases among people they know	“Yeah the only thing that would convince me not to go to a restaurant would be an outbreak at the restaurant…you still have to go out to get food or, or order out, but that would be the only thing that really would convince me if there was like a massive…outbreak.”–Participant 13, Focus Group 5, 18–29 year old female
Contact Tracing Apps	• Promote the app • Provide more information about the purpose of the app, how it works, privacy, etc. so individuals can make an informed choice about downloading and using it • Incentivize and reward use of the app (e.g., use gamification) • Make app use mandatory to enter some public places (e.g., stores, restaurants, workplaces)	“I think definitely really pushing…the fact that the app is not there to take any information from you, it’s not there to incriminate you, it’s not there to track your every movement; it’s there solely for the benefit of being able to create that web of figuring out where these hot spots are, or where its stemming from…I think that’s extremely beneficial.”–Participant 28, Focus Group 2, 18–29 year old male
Taking a Vaccine When Available	• Messaging needs to convey that a vaccine is safe, effective and backed by clear scientific evidence • Workplaces or other sectors (e.g., airlines) could make vaccines mandatory • Use peer influence	“I guess if I was forced into that, that it was the only way that I could travel and get back on an airplane again, then I would be kind of forced into it, because I need to be able to travel again for work.”–Participant 61, Focus Group 9, 30–59 year old female

## Discussion

This study addresses a critical need to further understand attitudes and current behaviours regarding, and barriers to uptake of, public health measures during the pandemic to inform public health messaging to change behaviour. Among a diverse group of 50 focus group participants, attitudes, current behaviours and barriers to following current public health measures were explored with respect to wearing face masks in public places, physical distancing, staying home when ill, avoiding high risk spaces like bars, using a contact tracing app and taking a vaccine when available. Four additional themes were identified with respect to public health measures. These themes were the importance of public health measures, ease of compliance with public health measures, critiques of public health messaging and suggestions for improving this messaging. Physical distancing and masking were consistently identified as more important than use of a contact tracing app for reducing transmission of COVID-19 while wearing a face mask was perceived to be the easiest to comply with. Participants felt that public health messaging to date has been conflicting and at times unclear, and provided suggestions on how to improve public health messaging including using framing like the responsibility of protecting others, and returning society to normal, and tailoring messaging strategies to specific population segments like younger people.

Framing has been used effectively in public health messaging and a recent systematic review on public health messaging around preventative behaviours like smoking cessation found that gain-framed messages were more effective than loss-framed messages [19]. Several participants in our study suggested that public health messaging could be improved by framing public health messaging around protecting others. Framing public health messaging using a moral frame focused on the responsibility of protecting others including family and friends has been shown to be an effective approach for influencing behaviours to mitigate COVID-19 transmission [20].

Consistent with the findings in this study, other studies have found that prosocial frames can be effective in influencing behavioural intentions with respect to COVID-19 public health measures [21–23]. One study [22] found that a prosocial frame emphasizing the public benefits of COVID-19 prevention behaviours was more effective than emphasizing personal benefits. Another study [21] looking at the effect of messaging interventions on willingness to engage in self-isolation found that prosocial messages were more effective than fear based ones.

A meta-analysis on the effectiveness of fear interventions found that perceived threat and perceived efficacy are important determinants of behaviour intention and behaviour change [24]. The authors found that high perceived threat and perceived efficacy (i.e., ability to make a change) were associated with greater changes in behavioural intention and actual behaviour; however, high perceived threat combined with low-efficacy was associated with defensive reactions or avoidance [24]. In the context of COVID-19, Carpraro and Barcelo [25] found that perceived threat to a person’s community was associated with increased behavioural intention to wear a face mask. This was echoed by participants in our study who noted that the perceived threat to the community, specifically to those that might get quite sick if they were to become infected, was motivation to wear a face mask in public places.

A recent study by Capraro and Barcelo [26] compared the effect of priming reasoning and priming emotion on the intention to wear a face mask to reduce the spread of COVID-19. The authors found that priming reasoning increased behavioural intention to wear a face mask, while priming emotion did not [26]. This is consistent with a public messaging theme identified in our study–it is important to provide a scientific rationale for why public health measures are necessary in order to improve adherence.

Gender differences have been identified with respect to perception of the threat of COVID-19 with more women believing they will be seriously affected by COVID-19 infection than men [25]. Intention to wear a face mask also varies by gender with women having greater intention than men to wear a face mask to mitigate the spread of COVID-19 [25]. These findings suggest that targeted messaging aimed at specific population segments may be required.

Participants also highlighted that public health messaging has been inconsistent throughout the pandemic, and many expressed a lack of trust in authorities as a result. Lack of trust in authorities was emphasized by participants as a barrier with respect to taking a COVID-19 vaccine. Trust in authorities has been consistently associated with adherence to public health measures [14, 27]. A recent survey involving 13,426 individuals from 19 countries found that willingness to take a COVID-19 vaccine was associated with higher trust in information provided by government sources [28]. As vaccine roll out expands globally, it will be important for government leaders and health officials to continue to build trust in order to encourage vaccine uptake.

This study has notable strengths. Using a quota system and a qualitative design, we were able to add to the quantitative surveys [28–33] done in this field and explore deeper, the attitudes, behaviours and barriers towards public health measures in a diverse population. This qualitative study is the first step in a large mixed-methods research program with the goal of understanding attitudes, behaviours, and barriers to COVID-19 public health measures in order to inform public health communications. The data from this program of research will help to inform strategies for influencing behaviour change across a range of population segments. Building on the findings from these focus groups, our research team of academics, government, industry, and marketing partners will develop and test creative concepts as part of a novel and data-driven public health messaging strategy to facilitate behaviour change in adherence to public health measures to reduce transmission of COVID-19.

There are also several study limitations. As is common with focus groups generally, participant selection bias must be considered in terms of the potential for self-selection bias, something that may have been exacerbated by the use of a virtual platform. There was a chance of self-selection bias where individuals who participated in a focus group may have been more adherent to public health measures than those who chose not to participate in a focus group. Social desirability bias should also be considered as persons may have said what they felt the focus group moderators wanted to hear, or if their views differed from others in the group, they may have been less inclined to share. Some of these limitations were minimized by having two or more targeted focus groups for certain targeted populations (e.g., three focus groups with participants aged 18 to 29 years; two for those aged 30 to 59 or 60+ years).

## Conclusion

Attitudes towards public health measures and current behaviours were variable amongst the focus group participants. Several barriers to following public health measures were identified including conflicting public health messaging. Framing the public health messaging carefully, taking into consideration different audiences, and providing the evidence for public health measures was suggested to increase efficacy. Participants also suggested that creative concepts should be targeted to specific population segments. As an important foundational step to effect improved adherence to public health measures, this work aimed to understand the characteristics, attitudes, and behaviours of the public and the perceived barriers to adhering to public health measures. The next step will be to use this information to develop effective public health messaging to encourage behaviour change in adherence to public health measures to reduce transmission of COVID-19.

## Supporting information

S1 File Focus group script and questions. (DOCX)

S2 File NVivo codebook. (DOCX)
