# Supplementary material for: Attitudes, current behaviours and barriers to public health measures that reduce COVID-19 transmission: A qualitative study to inform public health messaging
Source: PLoS One. 2021 Feb 19;16(2):e0246941. doi: 10.1371/journal.pone.0246941 (PMC7895406; doi:10.1371/journal.pone.0246941)
Supplement: S1 File — (DOCX) [file pone.0246941.s001.docx]

## Appendix 1: Focus Group Questions

**Focus Group Questions**

1. ***Based on survey results, we have/know that …………………….***

***Have short description of the two apps to share as needed:***

*ABTraceTogether (Alberta’s contact tracing app) and COVID Alert (Federal app, currently works only in Ontario) are smartphone based apps that work on iOS and Android phones. They support contact tracing by enabling notification of contacts that the person with COVID may not be aware of (e.g., contacts at a bar that you might not know) and can allow for faster contact notification.*

*Both the Alberta and Canada app use Bluetooth handshakes to identify interactions between phones within 2-metres. When a person is called by a public health contact tracer, they will be able to access information on your exposure to others who they will need to contact. For ABTraceTogether, privacy is safe as the only things stored by the app are phone numbers and how long you were in contact with others (e.g., 15 minutes, 3 hours, etc.). This information is automatically deleted after 21 days. These apps do not use GPS and so do not track peoples’ location. Information is stored within a highly secure Alberta Health Services server (the same way other health information is kept), and not by government.*

**What are your thoughts on using the mobile contact tracing app (Alberta’s *ABTraceTogether* or Canada’s *COVID Alert*) to prevent COVID-19?**

***Prompts***

- *What concerns do you have around the app? Privacy? Government surveillance / location tracking? Don’t understand how to use it? Technical issues like an old phone which isn’t compatible, the iPhone foreground issue, etc.)?*
- *What would convince you to use the app? Knowing your information is kept private & there is no location tracking? Benefit to the health of you and your loved ones?*
- *If people around you (family, friends, coworkers, etc.) were using an app, would you be more likely to use it? How much more likely?*

1. ***Based on survey results, this next question may be targeted to certain FGs (e.g. younger groups).***

**What are your thoughts on avoiding bars, pubs, nightclubs or lounges to prevent COVID-19?**

*Prompts*

- *What is your perception of the risk or safety of going to bars, pubs, nightclubs or lounges?*
- *Why do you go to bars? Feel they are safe, or the safety vs. benefit ratio is acceptable?*
- *What would convince you to go to bars less frequently?*

1. ***The survey results related to physical distancing suggests that ……………………***

**What are your thoughts on physical distancing to prevent COVID-19?**

*Prompts*

- *What is your understanding of what physical distancing is?*
- *Are there situations where you are more or less likely to practice physical distancing? Does it depend on who you are with?*
- *What would lead you to physical distance regularly? Is it more important from your perspective to do this to protect yourself or your loved ones?*

**(4) What are your thoughts on staying home when sick, even if only mildly sick (e.g., runny nose, mild cough, mild sore throat but no fever or trouble breathing) to prevent COVID-19?**

*Prompts*

- *What is your understanding with what sick is? Do you know that COVID-19 symptoms are more than just cough, fever, and trouble breathing but could also include just a runny nose?*
- *Why do you stay home or not stay at home while sick?*
  - *Inadequate sick leave policies at work?*
  - *Do not care about seriousness of COVID-19?*
- *What would lead you to stay at home while sick more often?*
  - *Better understanding of symptoms?*
  - *Other reasons?*

**(5) *Based on survey results, we have/know that …………………….***

**What are your thoughts on a vaccine for COVID-19 when it becomes available?**

*Prompts*

- *What concerns do you have around a vaccine? Safety? Side effects?*
- *What would convince you to get the vaccine? Benefit to the health of you and your loved ones?*
- *If people around you (family, friends, coworkers, etc.) were getting the vaccine, would you be more likely to get it? How much more likely?*

**(6) What are your thoughts on masking in public to prevent COVID-19?**

*Prompts*

- *In what situations are you more or less likely to wear a mask?*
- *Why do you or do you not wear a mask in public? What makes it difficult or easy to do?*
- *What would lead you to wear a mask more regularly? Protecting yourself? Protecting others?*
