# Supplementary material for: Attitudes, current behaviours and barriers to public health measures that reduce COVID-19 transmission: A qualitative study to inform public health messaging
Source: PLoS One. 2021 Feb 19;16(2):e0246941. doi: 10.1371/journal.pone.0246941 (PMC7895406; doi:10.1371/journal.pone.0246941)
Supplement: S2 File — (DOCX) [file pone.0246941.s002.docx]

## Appendix 2: NVivo Codebook

## Nodes

| Name | Description | Files | References |
| --- | --- | --- | --- |
| Assessment of public health measures | Discussion comparing public health measures: what are easiest vs most difficult to comply with, with are more and less important | 0 | 0 |
| Compliance with public health measures |  | 0 | 0 |
| Compliance different across age groups | Very mixed experiences and perceptions here, with some viewing older people as less compliant and others younger people | 1 | 4 |
| Easier to comply with | What are the easier public health measures for this age group/other group and why? | 5 | 11 |
| More challenging to comply with | What are the most challenging public health measures for this age group/other group and why? | 3 | 6 |
| Difficulty not touching things in some settings |  | 1 | 2 |
| Critiques |  | 0 | 0 |
| Contradictory policies and messages, messages that don't make sense |  | 3 | 3 |
| Measures should come from CMOH, not government |  | 1 | 1 |
| More or different use of resources |  | 2 | 5 |
| Importance |  | 0 | 0 |
| Less important | What they feel is less important re preventing spread | 2 | 5 |
| More important | What they feel is more important for preventing spread | 3 | 9 |
| Not enough info to assess |  | 1 | 1 |
| Other COVID public health measures | Comments or discussion about other public health measures not specifically asked about (e.g., contact tracing) | 6 | 10 |
| Elderly people perhaps shouldn't risk going out |  | 1 | 1 |
| Overarching comments |  | 0 | 0 |
| Anything to get back to more normal, including getting the economy going |  | 2 | 2 |
| Asymptomatic spread |  | 2 | 2 |
| COVID not serious, so question measures |  | 2 | 3 |
| Making measures mandatory |  | 2 | 2 |
| Realize these are based on our best guesses |  | 1 | 1 |
| Support some personal choice | A range of views on what this means exactly. Some seem to feel that there should be no public health measures that are mandatory; and many feel that people have to make their own assessments re the risks they are willing to tolerate. | 4 | 9 |
| Quotes |  | 3 | 10 |
| Effective public health messaging | How to convince people that public health measures work, and to comply | 0 | 0 |
| Messaging | The kinds of messaging that are likely to be effective | 0 | 0 |
| Emphasize protecting others, everyone |  | 2 | 2 |
| Evidence, science based |  | 2 | 2 |
| Less confusion |  | 2 | 3 |
| Problems with misinformation and-or conflicting info | This can make it difficult for people to make informed decisions | 4 | 6 |
| Quotes |  | 2 | 3 |
| Recommended strategies & tactics | Broader strategies to be used, such as increasing awareness, education, advocating, bylaws More specific kinds of tactics that are likely to be effective in improving understanding and/or changing behaviour | 0 | 0 |
| Advertising, advocacy, education, information |  | 2 | 6 |
| Give more explanation of the why behind the measures |  | 2 | 2 |
| More awareness |  | 1 | 2 |
| Bylaws and enforcement |  | 1 | 3 |
| Specific communication tactics to try |  | 0 | 0 |
| Use social media |  | 1 | 6 |
| Use visuals | Many things are in printing, but many people are visual | 3 | 3 |
| Won't be able to convince some people |  | 1 | 7 |
| Life during a pandemic | Overall views of some of the experiences people shared re living during a pandemic, including doing risk assessments of various activities, testing positive, etc. | 0 | 0 |
| Creates tension between people |  | 3 | 3 |
| Experience with testing positive |  | 1 | 2 |
| Important to have something to do |  | 1 | 1 |
| Need to protect people around us; don't hurt others |  | 1 | 1 |
| Not all that bad; some pluses |  | 1 | 1 |
| People also need to live their lives |  | 4 | 5 |
| Important to have something to do |  | 1 | 1 |
| Stress, anxiety, worry |  | 2 | 3 |
| Masks in public |  | 0 | 0 |
| Behaviour change | What might convince you/others to wear a mask in indoor public spaces? | 0 | 0 |
| If everyone else does |  | 1 | 1 |
| Making it mandatory | Having a bylaw, stores making it mandatory, enforcement | 3 | 12 |
| More information |  | 2 | 2 |
| Concerns or challenges |  | 0 | 0 |
| Costly for some |  | 1 | 2 |
| Don't work |  | 1 | 1 |
| Find wearing a mask difficult |  | 2 | 3 |
| Incentives outlined in the survey |  | 1 | 1 |
| Lack of consistency from town to town |  | 1 | 1 |
| Makes communication challenging for some |  | 1 | 1 |
| Not being worn properly, poor quality |  | 1 | 2 |
| Experiences to date | Including any situations where they wouldn’t wear a mask in public, now that there are bylaws in place [in many places] | 0 | 0 |
| Needing to convince others |  | 1 | 2 |
| Situations where people are less likely to wear a mask | Are there any situations when you probably would not likely wear a mask? | 0 | 0 |
| Don't wear a mask |  | 1 | 1 |
| Medically exempt | Medical condition, children who re neuro-atypical, etc. Also, experiences related to not being able to wear a mask, rude comments etc. | 4 | 5 |
| Outdoors |  | 6 | 17 |
| Some family situations | There was a comment that in some cultures, elders have a lot of influence and if they refuse to wear a mask at a large family gathering, than others won’t - out of respect | 2 | 2 |
| When eating or drinking in a public setting e.g., restaurant |  | 2 | 3 |
| When forget to bring it with me |  | 2 | 2 |
| Where it’s not mandatory, such as in small towns |  | 4 | 5 |
| Situations where people are more likely to wear a mask |  | 0 | 0 |
| Public spaces indoors |  | 5 | 10 |
| Schools |  | 1 | 1 |
| When it's not possible to physically distance |  | 2 | 7 |
| When its mandatory |  | 3 | 3 |
| Overall thoughts | Overall thoughts about wearing a mask in public, including understanding how and when to use a mask | 0 | 0 |
| Confusing information, actions by authorities | Re when you need to wear a mask, what types of masks are most effective, etc. | 3 | 9 |
| Disagree with being mandatory |  | 2 | 3 |
| Don't be rude-get offended- about not wearing a mask |  | 2 | 2 |
| Enforcement |  | 2 | 2 |
| False sense of security |  | 1 | 1 |
| Good idea, easy public health measure | Including that it doesn’t cost much, and that everyone can do it. | 8 | 20 |
| Hides faces-humour |  | 1 | 2 |
| Protect others, more than yourself |  | 7 | 20 |
| Support making it mandatory in public places |  | 4 | 11 |
| Quotes |  | 5 | 7 |
| Physical distancing |  | 0 | 0 |
| Behaviour change | What would lead you to physical distance regularly? | 0 | 0 |
| More cases in community would convince people |  | 1 | 1 |
| More information and education | E.g., that need to practice physical distancing even when wearing a mask. | 1 | 3 |
| Challenges and barriers |  | 0 | 0 |
| Busy places |  | 4 | 6 |
| Confusing or mixed messaging from officials |  | 1 | 3 |
| Family gatherings |  | 1 | 1 |
| Some people don't seem to care |  | 1 | 2 |
| Stores |  | 8 | 17 |
| Work |  | 2 | 2 |
| Experience to date | o Situations where people are more likely to practice it o Situations where people are less likely to practice it | 0 | 0 |
| Situations where people do distance | Situations where they or others are social distancing | 0 | 0 |
| At work |  | 2 | 2 |
| In public, including shops |  | 5 | 9 |
| Social gatherings with people outside bubble |  | 2 | 2 |
| With friends |  | 3 | 5 |
| Things that help | Being outdoors, places that have infrastructure (e.g., marks on the floor, plexiglass) | 2 | 5 |
| When concerned about others in social bubble | Some people described being very careful about the physical distancing, as they had others in their social bubble who they were concerned about | 1 | 1 |
| Situations where people do not distance | Situations where they or others are not physical distancing | 0 | 0 |
| Church |  | 1 | 1 |
| Other precautions in place | Use hand sanitizer, wear masks. | 3 | 3 |
| Outdoors |  | 3 | 3 |
| Schools |  | 1 | 3 |
| Some social gatherings |  | 2 | 2 |
| Within social bubble |  | 6 | 21 |
| Work |  | 2 | 4 |
| Overall thoughts and understanding |  | 0 | 0 |
| Depends on other people; can't do alone |  | 5 | 10 |
| Differences across cultures |  | 1 | 1 |
| Don't do |  | 1 | 1 |
| Easy to do |  | 4 | 5 |
| Lack of physical distancing in school settings |  | 1 | 1 |
| Makes sense; good idea | -Most seemed to understand physical distancing (i.e., knew that the distance was 2 meters, and how it helped to prevent spread) | 4 | 9 |
| Quotes |  | 3 | 3 |
| Pubs, bars, restaurants, private parties |  | 0 | 0 |
| Behaviour change | What might convince you/others to go to bars, etc. less frequently? | 0 | 0 |
| Difficult to stop some people, social contact is important |  | 3 | 10 |
| No peer pressure, but on same page with peer group | - some young people felt they were not swayed too much by peer pressure, but rather were aligned with people in their social bubble, who tend to have similar tolerance for risks | 1 | 3 |
| Peer pressure or influence |  | 1 | 2 |
| People getting sick, outbreak | - younger people [18-29] note that it’s hard to be concerned about going to restaurants, pubs or bars when you really don’t see anyone around you getting sick. - If there were more outbreaks traced to these places, then might re-assess | 2 | 5 |
| Experience to date | o Situations where people are more likely to go out to bars, etc. o Situations where people are less likely to go out to bars, etc. | 0 | 0 |
| Going less often than pre-COVID | - Some people in the middle-age group, and parents, describe still going out to restaurants & pubs, but far less often than before COVID | 2 | 3 |
| Situations where people are less likely to go out to bars, etc. |  | 0 | 0 |
| Lack of social distancing at the table | - Less likely to go to a restaurant or pub with friends, due to lack of social distancing possible at the table itself - and needing to have mask off when eating and drinking | 1 | 3 |
| Not part of their lifestyle |  | 2 | 2 |
| Public health measures not complied with | - Less likely to go to back to places, when they are concerned with the lack of compliance with public health guidance | 3 | 3 |
| Risk of contracting COVID-19 | - For some, they perceived the risk as just too great - Sometimes particularly cautious as they have a chronic health issue that puts them at risk, or have family members that do or who are elderly | 4 | 7 |
| Case numbers going up too much |  | 1 | 1 |
| When too crowded, e.g. at night | This often means avoiding going to places at night | 2 | 5 |
| Situations where people are more likely to go out to bars, etc. |  | 0 | 0 |
| Go out with people in my social bubble | Note that young people may have different definitions of social bubbles | 2 | 3 |
| Public health measures are followed | - People more likely to visit those establishments that are following public health guidelines - This held true across all age-groups | 9 | 29 |
| When able to sit outside on a patio |  | 3 | 4 |
| When experience no ill effects | Some described going out with friends to bars, etc. and not getting infected, and none of their friends have gotten infected, so don’t think of this as risky behaviour | 1 | 1 |
| When in a smaller town |  | 1 | 1 |
| When places are less busy | Choosing times where the pub or bar is less likely to be really crowded, etc. | 2 | 2 |
| Overall thoughts | Overall thoughts, including perceptions of risk vs safety | 0 | 0 |
| Confusion about public health guidelines |  | 1 | 2 |
| Represents a risk of contracting COVID | - Recognize there was a risk in going out to these places, but risk varied considerably - Restaurants & pubs were felt to be less risky places to visit than clubs & bars -Clubs and bars more popular with younger popn., who are also often less risk adverse | 6 | 17 |
| Different risks across these places | Bars and clubs were felt generally to be far riskier a place to go than pubs and restaurants | 4 | 6 |
| Individuals need to manage their own risk |  | 1 | 1 |
| People drinking alcohol tend to make poor choices |  | 1 | 1 |
| Some want to support these businesses, | They are concerned that they won’t survive the pandemic | 3 | 3 |
| Quotes |  | 2 | 6 |
| Staying at home when sick |  | 0 | 0 |
| Behaviour change | What would help to ensure that more people stay at home when sick? | 0 | 0 |
| Did not before COVID but do now |  | 3 | 8 |
| Hard to convince people to stay home |  | 1 | 2 |
| Experiences to date | o Situations where people are more likely to stay home [e.g., good sick leave policies; have a comfortable home] o Situations where people are less likely to stay home [e.g., self-employed, work in gig economy] | 0 | 0 |
| Situations where people are less likely to stay home |  | 0 | 0 |
| Limited or no sick leave |  | 2 | 5 |
| Might not know that sick |  | 1 | 1 |
| Need to get something done, go to appt., run errands |  | 4 | 6 |
| Family responsibilities | People described being in situations where they need to leave their house, when having some symptoms, if they need to help someone close to them (e.g., a parent, a child) | 1 | 2 |
| Situations where people are more likely to stay home |  | 0 | 0 |
| Don't have to worry about sick leave limitations |  | 1 | 1 |
| Even with mild symptoms | Cough, allergies, runny nose | 8 | 21 |
| Have others to support them (e.g., get groceries) |  | 1 | 1 |
| New symptoms |  | 1 | 2 |
| Staying home from school |  | 1 | 1 |
| To protect others |  | 6 | 9 |
| When feeling really sick |  | 1 | 4 |
| Overall thoughts | Understanding of what sick means re COVID-19 (i.e., symptoms to watch out for); and when to stay home | 0 | 0 |
| Availability of sick time |  | 1 | 2 |
| Confusion re public health guidance |  | 1 | 3 |
| Fear, scary |  | 1 | 1 |
| Hard to know if it's COVID; no clear defns. from authorities |  | 6 | 12 |
| Policies at schools and or daycares |  | 1 | 1 |
| Stigma if sneezes or coughs |  | 4 | 6 |
| Testing availability, with fast results, is important |  | 1 | 1 |
| Trust people to do the right thing |  | 1 | 1 |
| Quotes |  | 3 | 7 |
| Untitled |  | 0 | 0 |
| Tracing Apps |  | 0 | 0 |
| App use by businesses | Views on whether businesses should ask their clients to view this app, and whether this would affect people’s use of this business | 1 | 1 |
| Impact on behaviour |  | 0 | 0 |
| More likely to go in |  | 2 | 4 |
| Would not, or less likely, to go in | - | 2 | 3 |
| Potential benefits | - Idea of having some businesses require people to have app on phone [as described in survey] before they enter viewed positively by some | 4 | 5 |
| Potential challenges | - potential backlash, is that city people go to smaller towns where these rules [in bars & clubs] aren’t in place, which could potentially increase spread - some felt governments would need to be involved, before businesses would do this - others felt that businesses would need to hire security people to enforce this [Walmart in GP example] | 0 | 0 |
| Could cause backlash, security issues |  | 1 | 3 |
| Not practical |  | 2 | 3 |
| Behaviour change | What might convince you/others to use the app? | 0 | 0 |
| For vulnerable people | - A few people said that they would use the app to help protect vulnerable people they might be in contact with (e.g., young people talked about grandparents); 60+ persons talked about neighbours | 2 | 3 |
| If more people used it, including peers | - Some people, across age-groups, said they would download the app if more people were using it. - One young person specifically said, their age group is often driven by peer pressure | 6 | 6 |
| Incentives | - the idea of providing incentives to people to use the app, rather than making it mandatory, was perceived positively by a number of people in the 18-29 age group -they provided a few specific ideas re how to do this | 3 | 7 |
| Make it mandatory | - not many felt that the app should be made mandatory (e.g., to enter a particular establishment, to go to work at a particular place), but those that did tended to be in the 18-29 age group | 3 | 4 |
| More information, promotion, education | - Many people, across all age groups described needing more information to make an informed choice. - Big themes in 18-29 age group in particular - | 7 | 22 |
| Needs to be simple and easy to use | - this was raised primarily in the FG of parents - easy to download, or help provided [60+ primarily] - glitches, including battery draining issue, fixed | 2 | 6 |
| Help with downloading or using the app | - a few people mostly in the 60+ age group said they would need someone to help them download he app, and/or show them how to use it | 3 | 3 |
| IF problems fixed |  | 3 | 3 |
| Seeing people with COVID | - need to see people around you getting seriously ill or dying of COVID to even believe this is something that is going on around you [one young person] | 1 | 1 |
| Would not be influenced | - a few people said that nothing would really influence them to use the app, for a variety of reasons - only one such comment from 18-29 age group; so more common in older age groups | 4 | 8 |
| Benefits | What might be the benefits of using the app? e.g., if businesses were using the app, would you be more likely to go there? | 0 | 0 |
| Good idea, can see benefits |  | 6 | 10 |
| Benefits lots of people, keeps us safe | - some [mainly younger people] saw it as a good tool for preventing spread, meaning that it would benefit many - felt younger people would benefit most, as they tend to out more | 3 | 5 |
| Young people benefit | Young people who go out more, and may not be as careful, would benefit from the app. | 2 | 4 |
| Helpful to know if exposed, including helpful to own health |  | 3 | 9 |
| Helps with, speeds up tracing-tracking |  | 5 | 15 |
| Well designed |  | 2 | 2 |
| Concerns and challenges | Explanations both of why people have some concerns with the app, or not | 0 | 0 |
| Don't understand how to download it, how it works | -Few people described having difficulty downloading it -Some had questions about how it worked | 3 | 4 |
| Misconceptions about how it works | - Expectation that phone buzzes or notifies whenever in close contact with a COVID positive person - Others described that because of these misconceptions & lack of understanding about how it works, you’ll never get enough people using it to be useful | 5 | 6 |
| Getting people to use it | Majority of people need to use the app in order for it to be accurate. The people who go out more (i.e., young people) are not using it. | 5 | 10 |
| Government surveillance, tracking | -This is not as big an issue as privacy & security -A few feel “it’s too close to big brother watching you all the time”, but not many -Some concerns related to misconception about how the app works (i.e., that you are being tracked where you are at all times via GPS) | 4 | 7 |
| Heard of problems with the AB app | - Some people [in older age groups], had heard the app was not working properly, and this was a reason why some deleted it from their phones | 3 | 5 |
| Have to keep open, battery drain | - Phone has to be on with the app running for it to work. It drains the battery. - Big issue for young people, as they use their phones a lot so need to preserve battery. -Many described deleting the app, after experiencing problems with battery drainage | 4 | 19 |
| Legal aspect | - one person expressed concern about liability re contract tracing overall (i.e., concerned about having to go into isolation for 2 weeks) - so implied they’d rather not know they’d be in contact with someone who tested positive | 1 | 1 |
| No concerns or problems, including with privacy, surveillance, etc. | - A number of people, across all age groups, said they really didn’t have any concerns about the app - including with the privacy, security and surveillance issues | 5 | 14 |
| Privacy, security | -Many concerned about privacy and security issues -some wanted more info about privacy and security before making decision to download -No if few concerns by people 18-29 yr. old FG’s -Lots of concerns in 60+ FG’s | 7 | 27 |
| Experiences to date |  | 0 | 0 |
| Does not use |  | 0 | 0 |
| Did not know anything or very much about it | - Many, across all age groups, were not aware of the app or had heard very little about it | 7 | 10 |
| Do not feel at risk | - Don’t know anyone who’s been sick with it, so don’t feel at risk [18-29 yr. old’s] | 1 | 3 |
| Do not usually use apps |  | 2 | 3 |
| Does not go out much | - a number of people said they weren’t going out much, for a variety of reasons, so didn’t feel they would need it | 3 | 7 |
| Had downloaded it but then deleted it | - A few people had downloaded the app and deleted it due to privacy concerns, having to have it open all the time, etc. [parents group] | 1 | 3 |
| No cellphone, or does not have phone on, or with them | - some older people [60+], noted that they keep their phones off, only using them for emergencies - one younger person described keeping her phone in her bag, when at work, not with her | 0 | 0 |
| Not many cases where people live | - some people living outside Calgary or Edmonton felt the app wasn’t of interest, as they have few cases | 2 | 4 |
| Does use |  | 3 | 3 |
| Federal contact tracing app | - number of people seemed more aware of, and had downloaded the federal app, or were waiting for it to be active in AB and then planned to download it - older age groups [i.e., not the 18-29 age group] - some preferred the idea of a Canada-wide app, and wanted AB to get onboard | 5 | 11 |
| Quotes |  | 3 | 15 |
| Vaccine |  | 0 | 0 |
| Behaviour change | What might convince you/others to get the vaccine? | 0 | 0 |
| If confident it was safe and effective |  | 2 | 5 |
| If mandatory |  | 1 | 2 |
| More comfortable if others around got it |  | 1 | 2 |
| Nothing would convince |  | 3 | 4 |
| Protect others |  | 2 | 2 |
| Benefits |  | 0 | 0 |
| Get back to normal |  | 2 | 4 |
| Protect self and others |  | 5 | 7 |
| Concerns |  | 0 | 0 |
| Bad experiences in the past |  | 1 | 2 |
| Not confident that it will work, or fear of harm |  | 5 | 6 |
| Political climate around vaccine |  | 3 | 4 |
| Rushed development |  | 5 | 10 |
| Too new |  | 2 | 3 |
| Likelihood of getting the vaccine when available |  | 0 | 0 |
| Would get it | Many said they would get it as soon as it was available, saying that they trusted the government and the healthcare system that that it would be properly tested, etc. | 8 | 27 |
| Would not get it |  | 7 | 10 |
| Would wait and see | Generally don’t want to be an ‘early adopter’. Some want to see scientific evidence that it’s safe and/or effective. | 8 | 19 |
| Overall thoughts | General comments and comments about vaccines in general | 0 | 0 |
| Comments on the science of vaccines |  | 4 | 6 |
| COVID not serious enough to require a vaccine |  | 1 | 1 |
| General thoughts about vaccines, including flu shots | Some also shared their views about vaccines in general. Many talked about getting the flu shot every year, so would get the COVID vaccine as well | 4 | 7 |
| Good idea; should be effective |  | 3 | 4 |
| Should be mandatory |  | 2 | 4 |
| Quotes |  | 7 | 17 |
| Untitled |  | 0 | 0 |
